# Rapid Emergency Medicine Score: A novel prognostic tool for predicting the outcomes of adult patients with hepatic portal venous gas in the emergency department

**DOI:** 10.1371/journal.pone.0184813

**Published:** 2017-09-15

**Authors:** Chen-June Seak, David Hung-Tsang Yen, Chip-Jin Ng, Yon-Cheong Wong, Kuang-Hung Hsu, Joanna Chen-Yeen Seak, Hsien-Yi Chen, Chen-Ken Seak

**Affiliations:** 1 Department of Emergency Medicine, Lin-Kou Medical Center, Chang Gung Memorial Hospital, Taoyuan, Taiwan; 2 College of Medicine, Chang Gung University, Taoyuan, Taiwan; 3 Institute of Emergency and Critical Care Medicine, School of Medicine, National Yang-Ming University, Taipei, Taiwan; 4 Institute of Occupational Medicine and Industrial Hygiene, College of Public Health, National Taiwan University, Taipei, Taiwan; 5 Department of Emergency Medicine, Taipei Veterans General Hospital, Taipei, Taiwan; 6 Division of Emergency and Critical Care Radiology, Department of Medical Imaging and Intervention, Lin-Kou Medical Center, Chang Gung Memorial Hospital, Taoyuan, Taiwan; 7 Laboratory for Epidemiology, Department of Health Care Management, and Healthy Aging Research Center, Chang Gung University, Taoyuan, Taiwan; 8 Department of Urology, Lin-Kou Medical Center, Chang Gung Memorial Hospital, Taoyuan, Taiwan; 9 Sarawak General Hospital, Kuching, Sarawak, Malaysia; Azienda Ospedaliero Universitaria Careggi, ITALY

## Abstract

**Objective:**

This study aims to evaluate the performance of Rapid Emergency Medicine Score (REMS), Rapid Acute Physiology Score (RAPS), and Modified Early Warning Score (MEWS) in ascertaining the severity of illness and predicting the mortality of adult hepatic portal venous gas (HPVG) patients presenting to the emergency department (ED). This will assist emergency physicians (EPs) in risk stratification.

**Methods:**

Data for 66 adult HPVG patients who visited the EDs of 2 research hospitals between October 1999 and April 2016 were analyzed. REMS, RAPS, and MEWS were calculated based on data in the ED, and probability of death was calculated for each patient based on these scores. The ability of REMS, RAPS, and MEWS to predict group mortality was assessed by using receiver operating characteristic (ROC) curve analysis and calibration analysis.

**Results:**

The sensitivity, specificity, and accuracy for each scoring system were 92.1%, 89.3%, and 90.9% for REMS, 86.8%, 82.1%, and 84.8% for RAPS, and 78.9%, 89.3%, and 83.3% for MEWS respectively. In the ROC curve analysis, the areas under the curve for REMS, RAPS, and MEWS were 0.929, 0.877, and 0.856 respectively.

**Conclusion:**

Our study is the largest series performed in a population of adult HPVG patients in the ED. The results from this study demonstrate that REMS is superior in predicting the mortality of these patients compared to RAPS and MEWS. We therefore recommend that REMS be used for outcome prediction and risk stratification of adult HPVG in the ED.

## Introduction

Hepatic portal venous gas (HPVG) was first noted by Wolfe and Evans in a 1955 report on neonates with necrotizing enterocolitis [1]. Though very rare, this critically important radiological sign has traditionally been associated with life-threatening surgical conditions [2–4] and high mortality rates of 29% to 90% [2, 3, 5–7]. With the widespread use of modern diagnostic imaging technology, the number of patients found to have HPVG has been increasing [2–4, 8]. Studies on these patients revealed that surgical intervention was not always necessary for those whose HPVG had a benign etiology [3–5, 8]. Hence, risk stratification in the emergency department (ED) is crucial in assisting emergency physicians (EPs), surgeons, and intensivists identify critically ill HPVG patients and counsel family members about their prognoses. There is however still a lack of an established method to rapidly, easily, and accurately predict the outcomes of these patients.

In our previous study [8], we found that the Simplified Acute Physiology Score II (SAPS II), Acute Physiology and Chronic Health Evaluation II (APACHE II) score, and Sequential Organ Failure Assessment (SOFA) score can provide potentially valuable prognostic information in assessing illness severity and predicting mortality of adult HPVG patients presenting to the ED. However, these scoring systems, which are meant for the intensive care setting, require laboratory investigations [8, 9] and hence may not be feasible for rapid scoring in all EDs.

Some of the more feasible and favorable scoring systems used in the ED to quickly identify critically ill patients and predict hospitalization include the Rapid Emergency Medicine Score (REMS) [9–12], Rapid Acute Physiology Score (RAPS) [9, 13], and Modified Early Warning Score (MEWS) [12, 14]. To the best of our knowledge, there is no literature describing the application of these ED scoring systems in HPVG patients. This study thus evaluates their performance in ascertaining illness severity and predicting mortality of adult HPVG patients presenting to the ED.

## Material and methods

### Study design

This is a retrospective data longitudinal analysis conducted from October 1999 to April 2016.

### Study setting

The study was conducted in the EDs of two training and research hospitals, Linkou Chang Gung Memorial Hospital (3338 beds with approximately 15000 ED patients monthly in 2016) and Keelung Chang Gung Memorial Hospital (1098 beds with approximately 5800 ED patients monthly in 2016).

### Ethics with study approval

The study was performed following approval by Chang Gung Medical Foundation Institution Review Board (105-1133C).

### Data collection

Patients older than 18 years who were admitted to the ED with abdominal pain and had undergone a contrast-enhanced abdominal computed tomography (CT) scan with the findings of HPVG within this period were recruited. Initial keywords searched included ‘portal venous gas’, ‘portal gas (air)’, and ‘hepatic gas (air)’. Subsequently, a total of 66 HPVG patients were selected via a search engine by examining all radiological data stored in the hospitals’ emergency radiological database, with confirmation by a board-certified radiologist with over 20 years of experience in abdominal imaging. Medical records were carefully reviewed for the following parameters: age, sex, clinical presentation, temperature, heart rate, respiratory rate, blood pressure, Glasgow Coma Scale (GCS), and peripheral oxygen saturation. These parameters were assessed carefully alongside radiographic imaging and any other relevant data. All of the physiological scores were computed using these data. All of the patients in the study had a contrast-enhanced CT scan.

### Etiology

If surgery was performed, the intraoperative findings and histopathology reports were integrated and analyzed to confirm the underlying etiology. Clinical presentations, CT scan images, inpatient progress, and discharge diagnoses were considered as reference standards when surgery was not done. The study end-point was taken as mortality or survival upon discharge from our hospitals.

### Statistical analysis

Numerical and categorical variables are shown as mean±SD, and frequencies are displayed as percentages (%). Univariate analyses were applied to study the association between predictors and mortality, while categorical and numerical variables were analyzed with a chi-square test and two-sample t-test respectively. A logistic regression analysis was performed to develop predictive models between scoring systems and mortality. The probability of death was calculated based on the predictive models using the logit formula:

$$p=\frac{1}{1+\text{exp}\left[-\left(\beta_{0}+\beta_{1}X_{1}\right)\right]}$$

(*β*_0_: Intercept; *β*_1_: Parameter estimate of score; *X*_1_: Score)

A two-sample t-test was applied to compare differences in death probability between non-survivors and survivors. AUROC analysis was used to compare mortality predictability among scoring systems. Hosmer-Lemeshow Statistic was used to determine model fitness of risk prediction. Additionally, sensitivity, specificity, and rate of accuracy were generated based on the optimal cutoff point derived from the AUROC analysis.

## Results

A total of 66 patients who met the inclusion criteria were identified over 16.5 years. Among the 66 patients, 45 patients had ischemic bowel disease. In the group of 21 patients without ischemic bowel disease, 7 had intra-abdominal abscesses, 5 had biliary tract infections, 7 had colitis, 1 had gastric ulcer, and 1 had appendicitis.

All scoring systems were found to be significantly different between nonsurvivors and survivors. Statistically significant differences in patient variables are as follows: patient age of 73.47 years in nonsurvivors versus 63.46 years in survivors (p<0.05), mean arterial pressure of 63.62 mmHg in nonsurvivors versus 78.94 mmHg in survivors (p<0.05), respiratory rate of 25.53 breaths per minute in nonsurvivors versus 20.96 breaths per minute in survivors (p<0.05), Glasgow Coma Scale (GCS) of 9.08 in nonsurvivors versus 14.39 in survivors (p<0.0001), REMS of 14.21 in nonsurvivors versus 6.86 in survivors (p<0.0001), RAPS of 8.08 in nonsurvivors versus 3.11 in survivors (p<0.0001), and MEWS of 8.79 in nonsurvivors versus 4.43 in survivors (p<0.0001). All other patient characteristics were not significant different for both groups (Table 1).

**Table 1:** Comparison of findings between survivors and nonsurvivors.

Characteristic	Total n = 66	Nonsurvivors n = 38	Survivors n = 28	P
Age*	69.23 ± 16.64	73.47 ± 14.36	63.46 ± 18.00	0.0145
**Sex**				0.6991
**Female**	30 (45.45)	16 (53.33)	14 (46.67)	
**Male**	36 (54.55)	22 (61.11)	14 (38.89)	
Mean arterial pressure (mmHg)*	70.12 ± 31.02	63.62 ± 34.36	78.94 ± 23.62	0.0357
**Body temperature (°C)**	36.85 ± 1.67	36.70 ± 1.80	37.06 ± 1.47	0.3974
**Pulse rate (/min)**	112.30 ± 36.01	112.11 ± 44.43	112.57 ± 20.40	0.9547
Respiratory rate (/min)*	23.59 ± 9.26	25.53 ± 10.95	20.96 ± 5.45	0.0303
Glasgow Coma Scale*	11.33 ± 4.62	9.08 ± 4.87	14.39 ± 1.40	<.0001
REMS score*	11.09 ± 5.23	14.21 ± 4.01	6.86 ± 3.39	<.0001
RAPS score*	5.97 ± 4.15	8.08 ± 3.60	3.11 ± 2.99	<.0001
MEWS score*	6.94 ± 3.46	8.79 ± 2.90	4.43 ± 2.44	<.0001
**Type of management**				0.7241
**Conservative**	24 (36.36)	15 (62.50)	9 (37.50)	
**Surgery**	42 (63.64)	23 (54.76)	19 (45.24)	
**ED presentation to operation (hours)**	6.94 ± 3.46	7.51 ± 11.13	8.00 ± 7.80	0.8435
**Symptom onset to ED presentation (hours)**	49.98 ± 29.03	50.38 ± 31.11	49.45 ± 26.49	0.8983

* Indicates a statistically significant difference between the survivors and nonsurvivors.

Based on the predictive model with the scoring systems using logistic regression analysis, the probability of death was calculated and compared between nonsurvivors and survivors. Using REMS, the probability of death was 0.823 for nonsurvivors versus 0.240 for survivors (p<0.0001). With RAPS, probability of death was 0.755 in nonsurvivors versus 0.333 for survivors (p<0.0001). For MEWS, probability of death was 0.749 in nonsurvivors versus 0.341 in survivors (p<0.0001) (Table 2).

**Table 2:** The probability of death predicted by the REMS, RAPS, and MEWS scoring systems.

Assessment tools	Survivors	Nonsurvivors	P
**REMS** ^a^	0.240 ± 0.270	0.823 ± 0.220	<.0001
**RAPS** ^b^	0.333 ± 0.239	0.755 ± 0.229	<.0001
**MEWS** ^c^	0.341 ± 0.233	0.749 ± 0.254	<.0001

^a^ $\text{P}=\frac{1}{1+\text{exp}\left[-\left(-5.8633+0.5895*\text{REMS}\right)\right]}$

^b^ $\text{P}=\frac{1}{1+\text{exp}\left[-\left(-2.3268+0.5025*\text{RAPS}\right)\right]}$

^c^ $\text{P}=\frac{1}{1+\text{exp}\left[-\left(-3.0612+0.5176*\text{MEWS}\right)\right]}$

**Fig 1:**
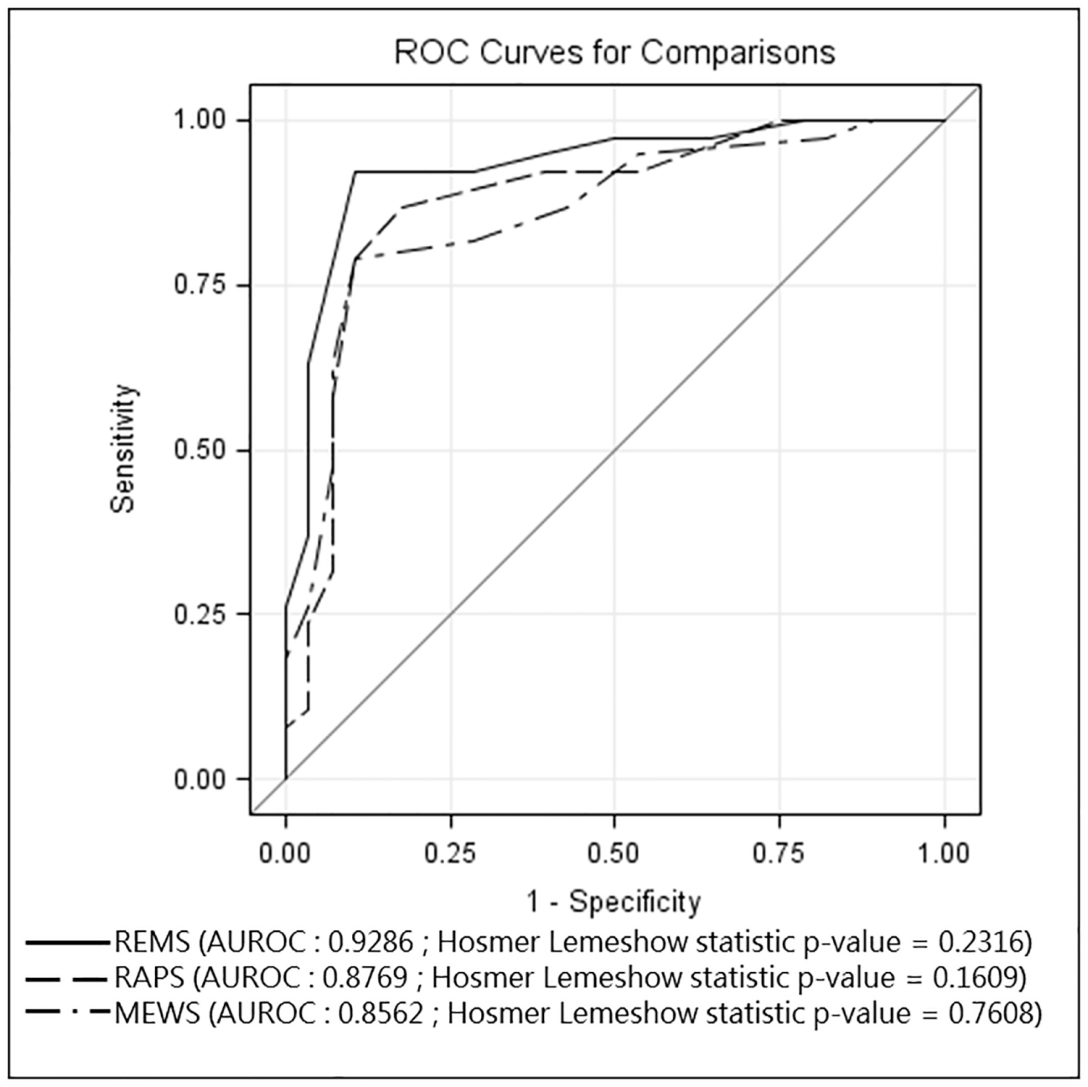
Receiver operating curves for predicting death according to REMS, RAPS, and MEWS.

The AUROC analysis demonstrated the predictability of REMS, RAPS, and MEWS as 0.9286, 0.8769, and 0.8562 respectively, while Hosmer Lemeshow statistic p-value were 0.232, 0.161, and 0.761 respectively (Fig 1).

The highest accuracy rate was found in the model with REMS (90.9%), compared with RAPS (84.8%) and MEWS (83.3%). REMS also had the highest sensitivity (92.1%) and specificity (89.3%) among the three scoring systems (Table 3).

**Table 3:** Sensitivity, specificity, and accuracy rates of the REMS, RAPS, MEWS scoring systems for predicting mortality.

Diagnostic values	REMS*	RAPS*	MEWS*
**Sensitivity**	35/38 (92.1%)	33/38 (86.8%)	30/38 (78.9%)
**Specificity**	25/28 (89.3%)	23/28 (82.1%)	25/28 (89.3%)
**Accuracy rate**	60/66 (90.9%)	56/66 (84.8%)	55/66 (83.3%)

*The optimal cutoff point of REMS, RAPS, and MEWS is 11, 4, and 6, respectively.

## Discussion

This study is to the best of our knowledge one of the largest analyses of adult HPVG patients in the ED. It is also the first report to date to assess clinical applicability of REMS, RAPS, and MEWS to these patients, in terms of determining illness severity and predicting mortality. A higher REMS, RAPS, and MEWS on ED admission were found to be a significant predictor of in-hospital mortality in adult HPVG patients despite the wide range of possible abdominal catastrophes.

Calculating REMS is a novel method to predict precisely yet easily the outcomes of adult HPVG patients in the ED. This scoring system was originally developed by Olsson et al based on a population of non-surgical ED patients [9–11]. As demonstrated in our study, REMS is superior in predicting the mortality of adult HPVG patients in the ED when compared to RAPS and MEWS. This adds to the evidence supporting the high adaptability of REMS—Olsson et al showed that it had the same predictive accuracy as the well-established but more complicated APACHE II score [10]. He also found that REMS was superior to RAPS as a powerful predictor of in-hospital mortality of ED patients with a wide range of common non-surgical disorders [11]. Furthermore, Goodacre et al showed that REMS is a better predictor of mortality than RAPS in emergency medical admissions [15], while Bulut et al demonstrated that REMS was more effective than MEWS in predicting hospitalization and in-hospital mortality of medical and surgical patients admitted to the ED [12]. Even in trauma patients, REMS also appears to be a simple, accurate predictor of in-hospital mortality [16].

REMS is calculated based on five physiologic parameters (mean arterial pressure, respiratory rate, heart rate, peripheral oxygen saturation, GCS score) and age [10, 11]. All of these measurements are always routinely collected and readily available in most ED setups, which makes REMS an easy-to-calculate and feasible tool in the ED setting. The AUROC for REMS of 0.9286 also illustrates its good discriminatory ability for predicting mortality in adult HPVG patients at time of ED presentation. In our patient population, REMS too performed the best in terms of sensitivity (92.1%) and specificity (89.3%) when predicting mortality. These results are important, as HPVG is only a radiologic finding that suggests a variety of underlying disease without providing a definite preliminary diagnosis [3, 4, 8, 17, 18], possibly confusing EPs, surgeons, and intensivists during practice. In addition to the high sensitivity and specificity of REMS, its accuracy of 90.9% can help EPs, surgeons, and intensivists to promptly identify critically ill HPVG patients, confidently stratify them more easily, and then discuss with family members regarding the prognosis and allocate the medical resources.

RAPS is an abbreviated version of the APACHE II score. It is highly predictive of mortality when calculated in the prehospital setting and extended to the full APACHE II score upon admission [9, 19]. As shown in our study, the specificity of RAPS of 82.1% was not as good as that of REMS and MEWS. A possible explanation is that RAPS is too abbreviated and only comprises heart rate, respiratory rate, mean arterial pressure and GCS. Since most of the variables included in RAPS are vital signs, the results of the score can easily be affected in anxious patients with non-specific abdominal pain caused by colitis, rather than suffering from life-threatening ischemic bowel disease.

MEWS is a simple, easily repeatable and non-disease-specific triage instrument used in the ED to predict mortality and thereafter decide between ward or intensive care unit admission [12, 20–23]. Although it has been found to be effective in predicting in-hospital mortality of medical and surgical patients presenting to the ED, its accuracy in predicting mortality of our patient population was not comparable to REMS or RAPS.

Reanalysis of statistical models after including surgery related variables did not produce statistically significant results with regards to predicting patient mortality. Such surgery related variables included type of management (conservative or surgery), duration between ED presentation and operation, and duration between symptom onset and ED presentation. This is because of the varied etiology of HPVG in our population, with 31.8% of the population not having ischemic bowel disease; the mortality of these patients without ischemic bowel disease depended on their clinical condition upon presentation, instead of surgery related variables. Another confounding factor affecting the association between surgery related variables and mortality was the reluctance of family members to provide consent on behalf of severely ill patients to undergo surgery. Nevertheless, these findings support our conclusion that REMS, RAPS, and MEWS were the dominant predictors of patient mortality, and that mortality rates can still be easily assessed based on the patient’s clinical state at time of presentation.

It should be acknowledged that the findings of our retrospective study require confirmation through a prospective study. Our study was also limited by the small population size, though it already is one of the largest analyses conducted in the population of adult HPVG patients presenting to the ED; this is due to the low incidence of HPVG hindering large-scale prospective studies.

## Conclusion

This study is the largest series performed in a population of adult HPVG patients presenting to the ED. Results demonstrated that REMS is superior in predicting the mortality of these patients, compared to RAPS and MEWS. We therefore recommend that REMS be used for outcome prediction and risk stratification in adult HPVG patients in the ED.

## Supporting information

S1 File Supporting information. (XLSX)

## References

[1] Wolfe JN, Evans WA. Gas in the portal veins of the liver in infants; a roentgenographic demonstration with postmortem anatomical correlation. *Am J Roentgenol Radium Ther Nucl Med*. 1955;74:486–8 13249015

[2] Monneuse O, Pilleul F, Barth X, Gruner L, Allaouchiche B, Valette PJ, et al Portal venous gas detected on computed tomography in emergency situations: surgery is still necessary. *World J Surg*. 2007;31:1065–71 doi: 10.1007/s00268-006-0589-0 17429565 10.1007/s00268-006-0589-0

[3] Seak CJ, Hsu KH, Wong YC, Ng CJ, Yen DH, Seak JC, et al The prognostic factors of adult patients with hepatic portal venous gas in the ED. *Am J Emerg Med*. 2014;32:972–5 doi: 10.1016/j.ajem.2014.05.016 25043627 10.1016/j.ajem.2014.05.016

[4] Abboud B, El Hachem J, Yazbeck T, Doumit C. Hepatic portal venous gas: physiopathology, etiology, prognosis and treatment. *World J Gastroenterol*. 2009;15:3585–90 doi: 10.3748/wjg.15.3585 19653334 10.3748/wjg.15.3585 PMC2721230

[5] Faberman RS, Mayo-Smith WW. Outcome of 17 patients with portal venous gas detected by CT. *Am J Roentgenol*. 1997;169:1535–8 9393159 10.2214/ajr.169.6.9393159

[6] Leibman PR, Patten MT, Manny J, Benfield JR, Hechtman HB. Hepatic-portal venous gas in adults: etiology, pathophysiology and clinical significance. *Ann Surg*. 1978;187:281–287 637584 10.1097/00000658-197803000-00012 PMC1396434

[7] Muscari F, Suc B, Lagarrigue J. [Hepatic portal venous gas: is it always a sign of severity and surgical emergency?]. *Chirurgie*. 1999;124:69–72 10193035 10.1016/s0001-4001(99)80045-2

[8] Seak CJ, Ng CJ, Yen DH, Wong YC, Hsu KH, Seak JC, et al Performance assessment of the Simplified Acute Physiology Score II, the Acute Physiology and Chronic Health Evaluation II score, and the Sequential Organ Failure Assessment score in predicting the outcomes of adult patients with hepatic portal venous gas in the ED. *Am J Emerg Med*. 2014;32:1481–4 doi: 10.1016/j.ajem.2014.09.011 25308825 10.1016/j.ajem.2014.09.011

[9] Hargrove J, Nguyen HB. Bench-to-bedside review: outcome predictions for critically ill patients in the emergency department. *Crit Care*. 2005;9:376–83 doi: 10.1186/cc3518 16137387 10.1186/cc3518 PMC1269432

[10] Olsson T, Lind L. Comparison of the Rapid Emergency Medicine Score and APACHE II in nonsurgical emergency department patients. *Acad Emerg Med*. 2003;10:1040–8 14525735 10.1111/j.1553-2712.2003.tb00572.x

[11] Olsson T, Terent A, Lind. Rapid Emergency Medicine score: a new prognostic tool for in-hospital mortality in nonsurgical emergency department patients. *J Intern Med*. 2004;255:579–87 doi: 10.1111/j.1365-2796.2004.01321.x 15078500 10.1111/j.1365-2796.2004.01321.x

[12] Bulut M, Cebicci H, Sigirli D, Sak A, Durmus O, Top AA et al The comparison of modified early warning score with rapid emergency medicine score: a prospective multicentre observational cohort study on medical and surgical patients presenting to emergency department. *Emerg Med J*. 2014;31:476–81 doi: 10.1136/emermed-2013-202444 23562988 10.1136/emermed-2013-202444

[13] Rhee KJ, Fisher CJ, Willitis NH. The Rapid Acute Physiology Score. *Am J Emerg Med*. 1987;5:278–82 3593492 10.1016/0735-6757(87)90350-0

[14] Dundar ZD, Ergin M, Karamercan MA, Ayranci K, Colak T, Tuncar A, et al Modified Early Warning Score and VitalPac Early Warning Score in geriatric patients admitted to emergency department. *Eur J Emerg Med*. 2016;23:406–12 doi: 10.1097/MEJ.0000000000000274 25919485 10.1097/MEJ.0000000000000274

[15] Goodacre S, Turner J, Nicholl J (2006). Prediction of mortality among emergency medical admissions. *Emerg Med J* 23:372–5. doi: 10.1136/emj.2005.028522 16627839 10.1136/emj.2005.028522 PMC2564087

[16] Imhoff BF, Thompson NJ, Hastings MA, Nazir N, Moncure M, Cannon CM. Rapid Emergency Medicine Score (REMS) in the trauma population: a retrospective study. *BMJ Open*. 2014;4:e004738 doi: 10.1136/bmjopen-2013-004738 24793256 10.1136/bmjopen-2013-004738 PMC4024603

[17] Kinoshita H, Shinozaki M, Tanimura H, Umemoto Y, Sakaguchi S, Takifuji K, et al Clinical features and management of hepatic portal venous gas: four case reports and cumulative review of the literature. *Arch Surg*. 2001;136:1410–4. 11735870 10.1001/archsurg.136.12.1410

[18] Shah PA, Cunningham SC, Morgan TA, Daly BD. Hepatic gas: widening spectrum of causes detected at CT and US in the interventional era. *Radiographics*. 2011;31:1403–13. doi: 10.1148/rg.315095108 21918051 10.1148/rg.315095108

[19] Rhee KJ, Baxt WG, Mackenzie JR, Burney RE, Boyle V, O’Malley RJ, et al Differences in air ambulance patient mix demonstrated by physiologic scoring. *Ann Emerg Med*. 1990;19:552–556. 2109959 10.1016/s0196-0644(05)82188-2

[20] Subbe CP, Davies RG, Williams E, Rutherford P, Gemmell L. Effect of introducing the Modified Early Warning score on clinical outcomes, cardio-pulmonary arrests and intensive care utilisation in acute medical admissions. *Anaesthesia*. 2003;58:797–802. 12859475 10.1046/j.1365-2044.2003.03258.x

[21] Ghanem-Zoubi NO, Vardi M, Laor A, Weber G, Bitterman H. Assessment of disease-severity scoring systems for patients with sepsis in general internal medicine departments. *Critical Care*. 2011;15:1–7. 10.1186/cc10102 PMC3219360 21401927

[22] Subbe CP, Kruger M, Rutherford P, Gemmel L. Validation of a modified Early Warning Score in medical admissions. *QJM*. 2001;94:521–6. 11588210 10.1093/qjmed/94.10.521

[23] Armagan E, Yilmaz Y, Olmez OF, Simsek G, Gul CB. Predictive value of the modified Early Warning Score in a Turkish emergency department. *Eur J Emerg Med*. 2008;15:338–40. doi: 10.1097/MEJ.0b013e3283034222 19078837 10.1097/MEJ.0b013e3283034222

